# The influence of time and irrigant refreshment on biofilm removal from lateral morphological features of simulated root canals

**DOI:** 10.1111/iej.13342

**Published:** 2020-08-27

**Authors:** T. C. Pereira, R. J. B. Dijkstra, X. Petridis, W. J. van der Meer, P. K. Sharma, F. B. de Andrade, L. W. M. van der Sluis

**Affiliations:** ^1^ Department of Dentistry Endodontics and Dental Materials Bauru School of Dentistry University of São Paulo Bauru Brazil; ^2^ Center for Dentistry and Oral Hygiene University Medical Center Groningen University of Groningen Groningen The Netherlands; ^3^ Department of Orthodontics University Medical Center Groningen University of Groningen Groningen The Netherlands; ^4^ Department of Biomedical Engineering University Medical Center Groningen University of Groningen Groningen The Netherlands

**Keywords:** biofilms, irrigation, optical coherence tomography, removal, sodium hypochlorite

## Abstract

**Aim:**

To evaluate the effect of irrigant refreshment and exposure time of a 2% sodium hypochlorite solution (NaOCl) on biofilm removal from simulated lateral root canal spaces using two different flow rates.

**Methodology:**

A dual‐species biofilm was formed by a Constant Depth Film Fermenter (CDFF) for 96 h in plug inserts with anatomical features resembling an isthmus or lateral canal‐like structures. The inserts were placed in a root canal model facing the main canal. NaOCl 2% and demineralized water (control group) were used as irrigant solutions. Both substances were applied at a flow rate of 0.05 and 0.1 mL s^−1^. The samples were divided into three groups with zero, one or two refreshments in a total exposure time of 15 min. A three‐way analysis of variance (anova) was performed to investigate the interaction amongst the independent variables and the effect of consecutive irrigant refreshment on percentage of biofilm removal. A Tukey *post hoc* test was used to evaluate the effect of each independent variable on percentage biofilm removal in the absence of statistically significant interactions.

**Results:**

For the lateral canal, NaOCl removed significantly more biofilm irrespective of the number of refreshments and exposure time (*P* = 0.005). There was no significant effect in biofilm removal between the consecutive irrigant refreshments measured in the same biofilm. For the isthmus, NaOCl removed significantly more biofilm irrespective of the number of refreshments and exposure time; both NaOCl and a high flow rate removed significantly more biofilm when the exposure time was analysed (*P* = 0.018 and *P* = 0.029, respectively). Evaluating the effect of consecutive irrigant refreshment on the same biofilm, 2% NaOCl, 0.1 mL s^−1^ flow rate and one or two refreshments removed significant more biofilm (*P* = 0.04, 0.034 and 0.003, <0.001, respectively).

**Conclusions:**

In this model, refreshment did not improve biofilm removal from simulated lateral root canal spaces. NaOCl removed more biofilm from the lateral canal‐ and isthmus‐like structure. A higher flow rate removed significantly more biofilm from the isthmus‐like structure. There was always remaining biofilm left after the irrigation procedures.

## Introduction

Sodium hypochlorite (NaOCl) is the irrigant of choice during root canal treatment (Zehnder 2006, Dutner *et al*.** 2012). It has gained its popularity mainly due to its action against microorganisms (McDonnell & Russell 1999) and biofilm (Arias‐Moliz *et al*.** 2009, Bryce *et al*.** 2009) as well as its capacity to dissolve pulp tissue (Sirtes *et al*.** 2005) and organic components of the smear layer (Baumgartner & Mader 1987). However, NaOCl does not dissolve inorganic tissue (Sen *et al*.** 1999) and is not able to fully penetrate the biofilm (van der Waal *et al*.** 2014, 2017, Petridis *et al*.** 2019a) especially when the biofilm is densely packed with bacteria (Busanello *et al*.** 2019, Petridis *et al*.** 2019a, Petridis *et al*.** 2019b). Furthermore, biofilms withstand NaOCl treatment (Stewart *et al*.** 2001), which is also corroborated in *in situ* investigations of root canal specimens (Nair *et al*.** 2005, Ricucci & Siqueira 2010). Taking into account that biofilm remaining post‐treatment can re‐grow (Chávez de Paz *et al*.** 2008, Shen *et al*.** 2010, 2016, Ohsumi *et al*.** 2015), failure of apical periodontitis to resolve is an imminent risk. Therefore, achieving a better understanding of the properties of NaOCl is warranted in order to improve its antibiofilm efficacy.

NaOCl reacts by direct contact between free available chlorine molecules and organic matter (Moorer & Wesselink 1982). The flux of molecules will take place through diffusion or convection, with diffusion being slower than convection (Verhaagen *et al*.** 2014). The surface contact between irrigant–biofilm as well as the volume of irrigant will directly influence the availability of the free chlorine molecules to the biofilm. In the highly complex root canal system, the surface contact between irrigant–biofilm is limited, especially for lateral morphological features such as isthmuses and lateral canals. Moreover, it is not known whether the volume of irrigant present in the root canal contains sufficient free chlorine to allow for diffusion into the biofilm present in lateral morphological features.

The concentration of NaOCl will determine the amount of free chlorine. Clinically, concentrations ranging between 1 and 5% are commonly used (Dutner *et al*.** 2012). Refreshment is widely thought to be an effective method of compensation for the loss of chemical effectiveness of a lower concentration of NaOCl (Moorer & Wesselink 1982, Zehnder 2006). However, this has never been proven. Using a numerical diffusion model to predict the efficacy of diffusion, it has been advised to constantly apply fresh irrigant alongside the opening of an isthmus or lateral canal‐like structure to enhance diffusion (Verhaagen *et al*.** 2014). It has also been demonstrated that lower concentration NaOCl solutions have a significantly less effective reaction rate compared to high concentration solutions, even after multiple refreshments of the former (Macedo *et al*.** 2014a). Moreover, a constant flow of a 2% NaOCl using syringe irrigation did not remove more biofilm from lateral morphological features than an inert control solution (Pereira et al. 2020b ). In addition, within 30 s of exposure, increasing NaOCl concentration did not significantly improve the removal of biofilm from lateral morphological features (Pereira et al. 2020a).

Taking all the above into consideration, it is interesting to explore whether refreshment of a NaOCl solution of clinically relevant concentration applied at different flow rates and for clinically relevant application times could enhance biofilm removal from lateral morphological features in the root canal. Hence, the aim of this study was to evaluate the effect of 2% NaOCl refreshments, exposure time and flow rate on its capacity to remove biofilm from simulated lateral canal and isthmus‐like structures of the root canal using an *in vitro* root canal model.

The null hypothesis was that refreshment, exposure time and irrigant will not make a significant difference in removing biofilm from lateral morphological features from the simulated root canal.

## Material and methods

### Bacterial strains and growth conditions

A single colony of *Streptococcus oralis* J22 and *Actinomyces naeslundii* T14V‐J1 grown on blood agar plates was used to inoculate 10 mL modified brain heart infusion broth (BHI; Oxoid Ltd., Basingstoke, UK) (37.0 g L^−1^ BHI, 1.0 g L^−1^ yeast extract, 0.02 g L^−1^ NaOH, 0.001 g L^−1^ vitamin K1, 5 mg L^−1^ L‐cysteine‐HCl, pH 7.3) as previously described (Busanello *et al*.** 2019, Petridis *et al*.** 2019). The pre‐cultures were stored separately for 24 h in ambient air for *S. oralis* and in anaerobic chamber for *A. naeslundii*. Subsequently, bacteria were harvested by centrifugal force (6350 ***g***) and washed twice in sterile adhesion buffer (0.147 g L^−1^ CaCl_2_, 0.174 g L^−1^ K_2_HPO_4_, 0.136 g L^−1^ KH_2_PO_4_, 3.728 g L^−1^ KCl, pH 6.8). The bacterial pellets were suspended in 10 mL sterile adhesion buffer and sonicated intermittently in ice‐water for 3 × 10 s at 30 W (Vibra cell model 375; Sonics and Materials Inc., Newtown, CT, USA) to break bacterial chains. Following this, bacteria were counted using a Bürker‐Türk counting chamber (Marienfeld‐Superior, Lauda‐Königshofen, Germany) and the suspensions were diluted in 250 mL of adhesion buffer in a concentration of 6 × 10^8^ bacteria mL^−1^ for *S. oralis* and 2 × 10^8^ bacteria mL^−1^ for *A. naeslundii* in order to obtain the dual‐species bacterial suspension.

### Specimen preparation

**Figure 1:**
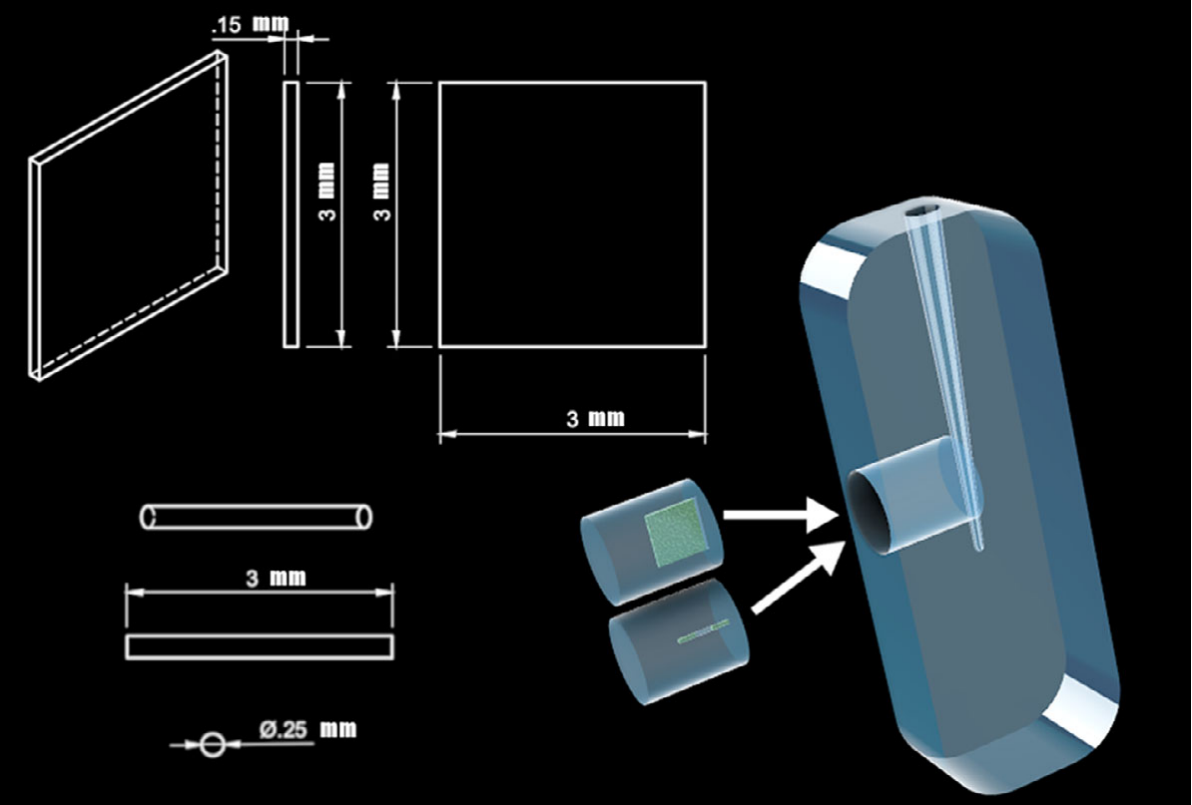
Schematic drawing of the artificial root canal and the plugs of isthmus‐like and lateral canal‐like structures.

Transparent PolyDiMethylSiloxane (PDMS) (PolyDiMethylSiloxane; Sylgard 184, Dow‐Corning, Midland, MI, USA) root canal models, with a small plug (*R* = 2.5 mm) in the apical area perpendicular to the root canal, were created using a size D finger spreader (Dentsply Sirona, Ballaigues, Switzerland) as described in Macedo *et al*.* *(2014b). The PDMS plug inserts with anatomical features resembling an isthmus or lateral canal were created using moulds containing a thin metal strip (width 3 mm, thickness 0.15 mm, length 3 mm, total volume 1.35 mm^3^) or a small cylinder (length 3.0 mm and thickness 0.25 mm, total volume 0.29 mm^3^), respectively (Fig. 1).

### Constant depth film fermenter biofilm

Steady‐state biofilms were grown using a constant depth film fermenter (CDFF) in which a constant dropwise supply of nutrients combined with a repeated cycle of compression/scraping leads to a dental plaque‐like bacterial dense biofilm (Kinniment *et al*.** 1996, Rozenbaum *et al*.** 2019). Lateral canal and isthmus inserts were coated with reconstituted human saliva. Briefly, whole human saliva was pooled from at least 20 volunteers of both genders (saliva was collected in agreement with the guidelines set out by the Medical Ethical Committee of the University Medical Centre Groningen, Groningen, The Netherlands, approval letter 06‐02‐2009) and freeze‐dried. Next, it was dissolved in 20 mL adhesion buffer (1.5 g L^−1^), stirred for 2 h and centrifuged at 10,000 × ***g***, at 10 °C, for 5 min. Both inserts were conditioned with the reconstituted saliva by dropwise inoculation in the CDFF at a constant low platform rotation that was stopped after 1 h. Samples were incubated with the saliva under static conditions for 14 h. After this, a 250 mL dual‐species bacterial suspension was introduced in the CDFF over 1 h at a constant slow rotation of the CDFF table. Rotation was stopped, and bacteria were allowed to adhere for 1 h to the saliva‐coated inserts. Subsequently, rotation was resumed and continuous supply of modified BHI (45 mL h^−1^) was started so biofilms could growth over the next 96 h at 37 °C.

### Irrigation protocols and OCT analysis

**Figure 2:**
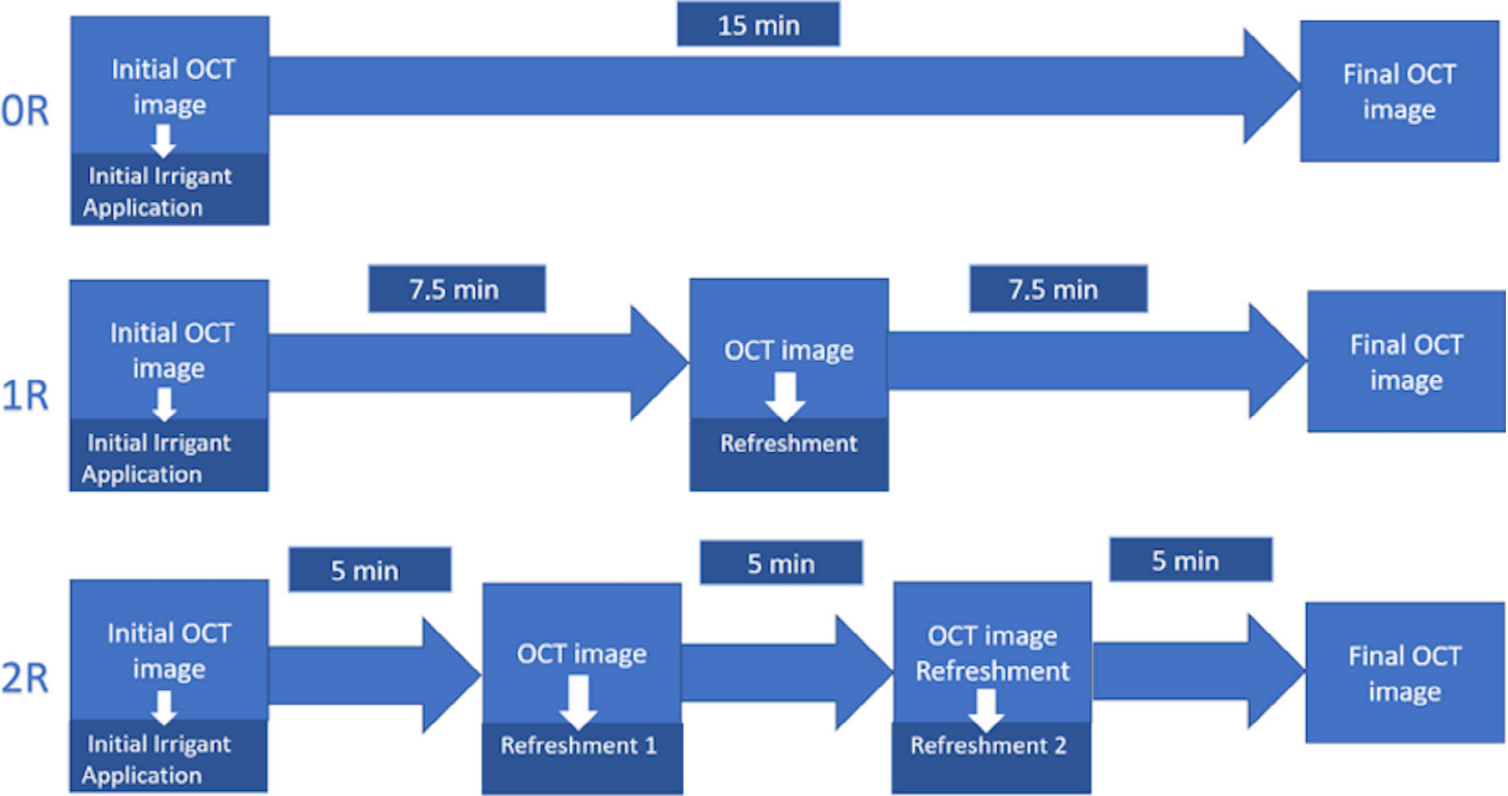
Schematic representation of the experimental groups.

After 96 h, the inserts with lateral canal‐ and isthmus‐like structures filled with biofilm were removed from the CDFF, carefully placed in the root canal model and analysed using optical coherence tomography (Thorlabs Ganymede II, Newton, NJ, USA). The purpose of these OCT scans was to determine the initial biofilm volume present (pre‐treatment scan). The experimental groups were established based on an irrigation procedure of 15 min with an irrigant solution (NaOCl or sterile demineralized water‐control), applied at 2 different flow rates (0.05 or 0.1 mL s^−1^) and different number of refreshments (0, 1 or 2). The 120 samples of lateral canal and 120 samples of isthmus‐like inserts were randomly divided into 12 experimental groups (*n* = 10). Six groups were irrigated with demineralized water and the other six with 2% NaOCl. For each irrigant, three groups were irrigated at 0.05 mL s^−1^ and the other three at 0.1 mL s^−1^. For each irrigant and flow rate, three different protocols were used: no refreshment (0R), 1 refreshment at 7.5 min (1R) and 2 refreshments at 5 and 10 min (2R). In all the groups, OCT scans were made after the irrigation procedure and before each refreshment (post‐treatment scan) (Fig. 2). In the group with 2 refreshments, the percentage of biofilm removal after each step was evaluated (consecutive irrigant refreshment).

The 2% NaOCl concentration was obtained from a standard solution of NaOCl 12–15% (Sigma‐Aldrich, St Louis, MO, USA) by iodometric titration before every experiment. The irrigation process was performed with a 5‐mL irrigation syringe (Ultradent Products Inc.) and 30G irrigation needle (Endo‐Eze; Ultradent Products Inc., South Jordan, UT, USA). During irrigation, the needle was placed 2 mm coronal to the apical end‐point of the root canal. In the 0.05 mL s^−1^ flow rate groups, 1.5 mL of the irrigating solution was released over 30 s by in and outward movements of 5 mm amplitude. In the 0.1 mL s^−1^ flow rate groups, the 1.5 mL of the irrigants was released in 15 s.

### OCT image analysis

The software program ImageJ FIJI (National Institutes of Health, Bethesda, MD, USA) was used for the quantitative image analyses. By acquiring 3D scans, containing 750 slices of 750 × 373px (field of view 5.0 mm, refractive index 1.33), the volume of biofilm residing inside the isthmus or lateral canal‐like structures could be determined. All 750 slices per sample were analysed based on their greyscale composition, and by thresholding to only select the biofilm and filtering the background noise. Percentage biofilm removal was calculated by determining the difference in biofilm volume between pre‐treatment scans and post‐treatment scans after the refreshments and after the 15 min of irrigant exposure.

### Statistical analysis

Statistical analysis was performed using SPSS software (version 24.0; IBM Corp., Armonk, New York, USA). A three‐way analysis of variance (anova) was performed to investigate the interaction amongst the three independent variables, namely *irrigant* (demineralized water, 2% NaOCl), *flow rate* (0.05, 0.1 mL s^−1^) and *exposure time* (5, 7.5, 15 min) or *irrigant* (demineralized water, 2% NaOCl), *flow rate* (0.05, 0.1 mL s^−1^) and *irrigant refreshment* (0, 1, 2) on *percentage biofilm removal* (dependent variable). In the absence of statistically significant interactions, a main effect analysis was carried out in order to investigate the effect of each independent variable on percentage biofilm removal (Tukey *post hoc* test). A three‐way mixed anova was performed in order to investigate the effect of consecutive irrigant refreshment on percentage biofilm removal, whereby *irrigant* (demineralized water, 2% NaOCl) and *flow rate* (0.05, 0.1 mL s^−1^) were the between‐subjects independent variables and the *consecutive irrigant refreshments of the same biofilm* in the group with 2 refreshments the within‐subjects independent variable. In the absence of statistically significant interactions, a main effect analysis was carried out in order to investigate the effect of each independent variable on percentage biofilm removal (Tukey *post hoc* test).

## RESULTS

### Lateral canal

#### Chemical effect of the irrigant on biofilm removal

**Figure 3:**
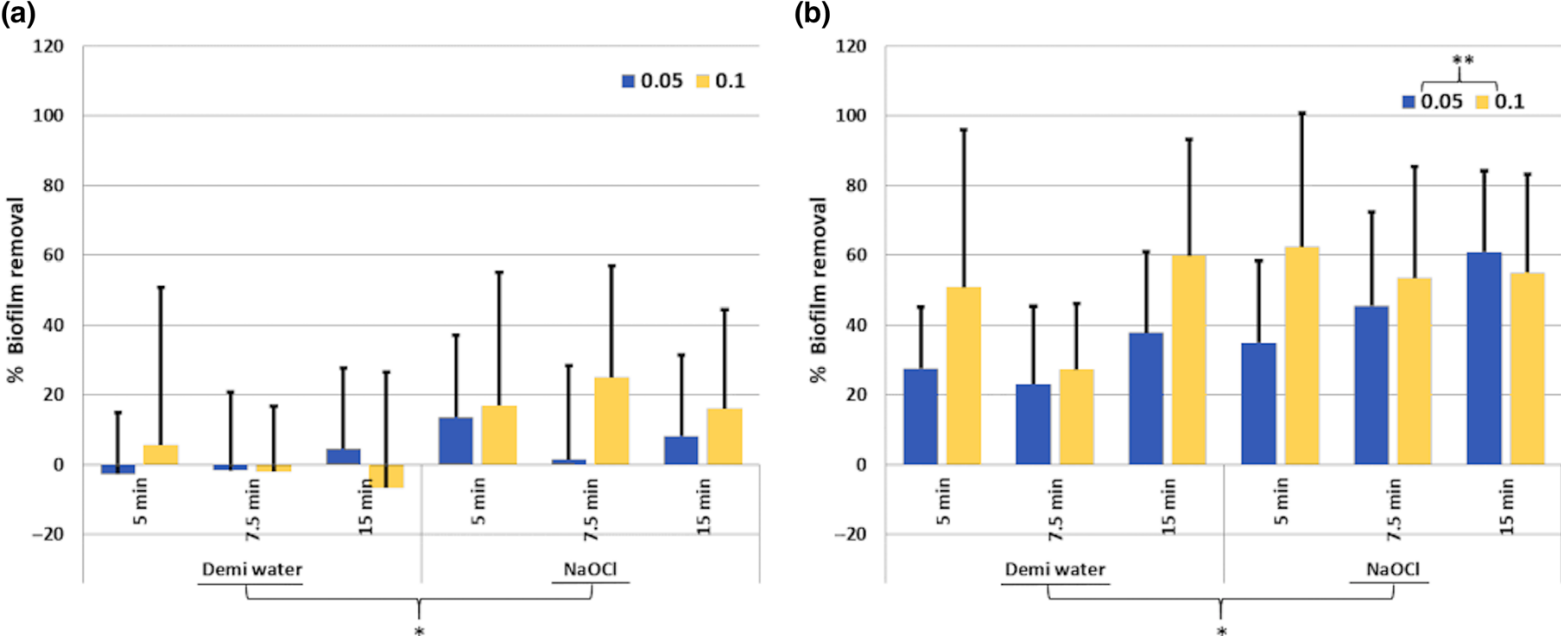
Chemical effect of an irrigant (demineralized water, 2% NaOCl) after 3 different exposure times (5, 7.5 or 15 min) applied with either flow rate 0.05 mL s^−1^ (dark grey) or 0.1 mL s^−1^ (light grey) on biofilm removal (%) from a lateral canal (a) or isthmus (b) like structure. * Indicates significant difference between the irrigants, whereas ** Indicates a significant difference for the flowrate (mL s^−1^).

Three‐way anova revealed no significant interaction amongst the independent variables on percentage biofilm removal. The main effect analysis revealed no significant effect of exposure time and flow rate on biofilm removal. The main effect of irrigant on biofilm removal was significant (*P* = 0.005) with 2% NaOCl removing significantly more biofilm than demineralized water irrespective of the exposure time and flow rate (Fig. 3).

#### Effect of irrigant refreshment on biofilm removal measured after 15 min

**Figure 4:**
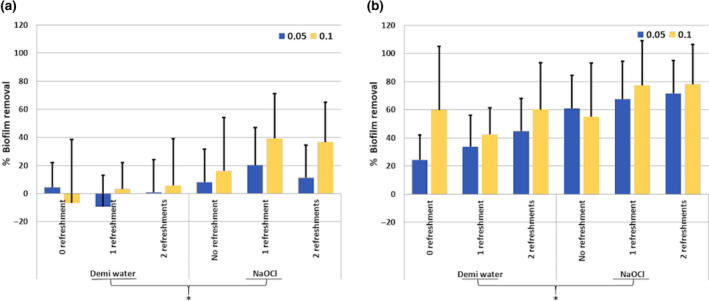
The influence of the number of refreshments of demineralized water or 2% NaOCl applied with either flow rate 0.05 mL s^−1^ (dark grey) or 0.1 mL s^−1^ (light grey) on biofilm removal (%) after a total exposure time of 15 min from a lateral canal‐ (a) or isthmus (b)‐like structure. Significant difference between the irrigants is indicated by an *.

Three‐way anova revealed no significant interaction amongst the independent variables on percentage biofilm removal. The main effect analysis revealed no significant effect of refreshment and flow rate on biofilm removal. The main effect of irrigant on biofilm removal was significant (*P* < 0.001), with 2% NaOCl removing significantly more biofilm than demineralized water irrespective of refreshment and flow rate (Fig. 4).

### Effect of consecutive irrigant refreshment of the same biofilm on biofilm removal

**Figure 5:**
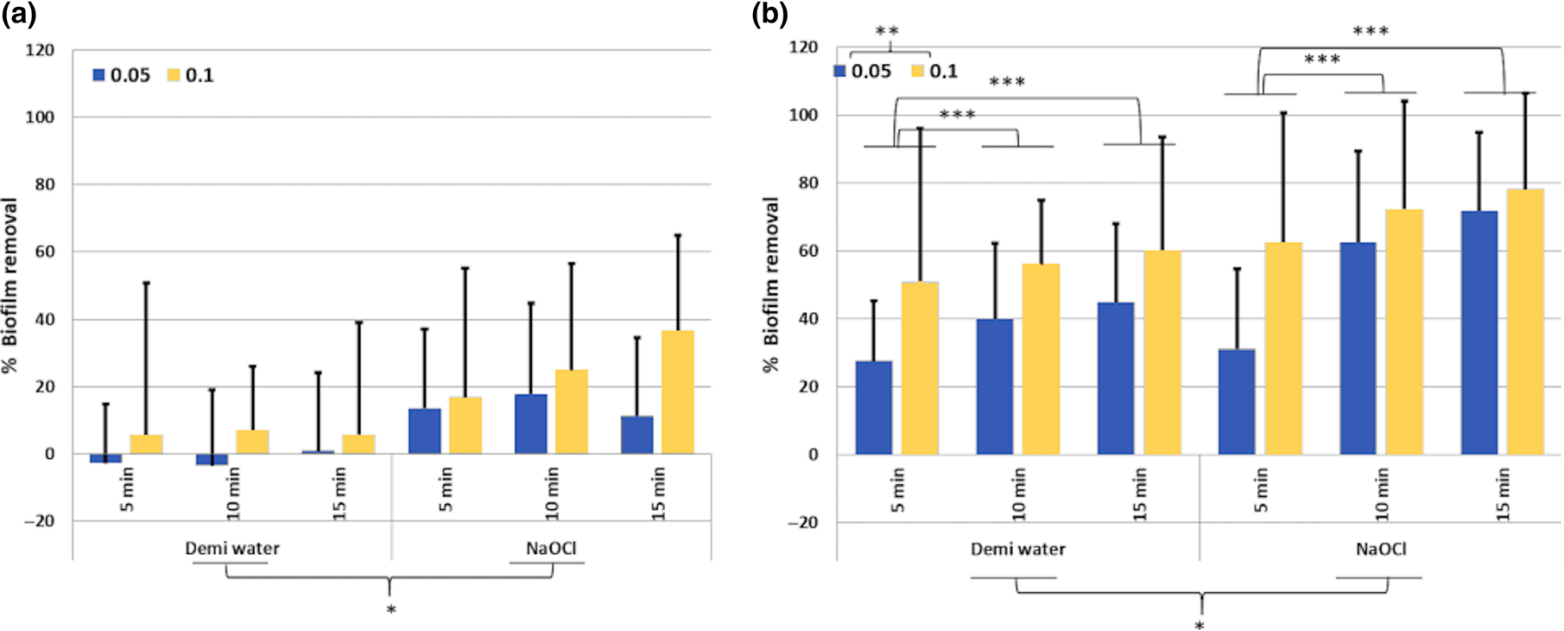
The influence of consecutive irrigation steps (5, 10 and 15 min) using demineralized water or 2% NaOCl, applied with either flow rate 0,05 mL s^−1^ (dark grey) or 0.1 mL s^−1^ (light grey) on biofilm removal (%) from a lateral canal‐ (a) or isthmus (b)‐like structure. Significant difference between the irrigants is indicated by an * whereas ** Indicates the significant difference for the flowrates (mL s^−1^). *** Shows significant influence between the different irrigation steps.

Three‐way mixed anova revealed no significant interaction amongst the independent variables on percentage biofilm removal. The main effect analysis revealed no significant effect of consecutive irrigant refreshment and flow rate on biofilm removal. The main effect of irrigant on biofilm removal was significant (*P* = 0.018), with 2% NaOCl removing significantly more biofilm than demineralized water irrespective of consecutive irrigant refreshment and flow rate (Fig. 5).

### Isthmus

#### Chemical effect of the irrigant

Three‐way anova revealed no significant interaction amongst the independent variables on percentage of biofilm removal. The main effect analysis revealed no significant effect of exposure time on biofilm removal. The main effects of irrigant and flow rate on biofilm removal were significant (*P* = 0.018 and *P* = 0.029, respectively), with 2% NaOCl and 0.1 mL s^−1^ removing significantly more biofilm irrespective of the exposure time (Fig. 3).

#### Effect of irrigant refreshment on biofilm removal measured after 15 min

Three‐way anova revealed no significant interaction amongst the independent variables on percentage biofilm removal. The main effect analysis revealed no significant effect of refreshment and flow rate on biofilm removal. The main effects of irrigant on percentage biofilm removal was significant (*P* < 0.001), with 2% NaOCl removing significantly more biofilm irrespective of refreshment and flow rate (Fig. 4).

#### Effect of consecutive irrigant refreshment on biofilm removal

Three‐way anova revealed no significant interaction amongst the independent variables on percentage biofilm removal. The main effect analysis revealed no significant effect of flow rate on biofilm removal. The main effects of irrigant, flow rate and consecutive irrigant refreshment on percentage biofilm removal were significant (*P* = 0.04, 0.034 and 0.003, <0.001) with respectively 2% NaOCl, 0.1 mL s^−1^ and one or two refreshments removing more biofilm (Fig. 5).

## Discussion

In this study, a root canal model with lateral morphological features filled with biofilm was used and the outcome measure was percentage biofilm removal evaluated by optical coherence tomography (OCT). OCT is unique in the sense that it enables a longitudinal evaluation of biofilm without the need for prior staining. This allows for measurements before and after each irrigation step. This implies that each biofilm is its own control. This is important in biofilm research given that biofilm growth is difficult to standardize even when using standardized laboratory procedures (Swimberghe *et al*.** 2019). A bacterial dense dual species *in vitro* biofilm (*S oralis* and *A. naeslundii*) was used due to its strong collateral bonds improving biofilm cohesion and adhesion (He *et al*.** 2013, Busanello *et al*. 2019). In an earlier study using this typical model, biofilm removal highly correlated with the irrigant flow pattern and streaming velocities (Pereira et al. 2020a). Furthermore, the bacteria comprising this biofilm are often found in infected root canals (Chávez de Paz *et al*.** 2003), the biofilm adheres to PDMS (Song *et al*.** 2015) and its viscoelastic properties resemble closely those of an *in vivo* oral biofilm (He *et al*.** 2013). Importantly, considering that an isthmus is located between two canals, it is possible to hypothesize that during irrigation, a continuous flow of the irrigant solution between the two canals would remove more biofilm. However, isthmuses have an irregular shape and many isthmuses are not completely open; furthermore, during instrumentation of the root canal, dentine debris is packed in the isthmuses potentially closing the open structure (Robinson *et al*.** 2013). The root canal model used in the present study is a closed system that hampers the biofilm removal when compared to the removal from an open isthmus between two canals.

The negative values in Figures 3,4 and 5, related to the lateral canal‐like structure, indicate biofilm expansion. The fluid flow produced during syringe irrigation can induce wall shear stress, possibly leading to a mechanical disruption of biofilm causing absorption of energy into the biofilm resulting in volumetric expansion (Busscher *et al*.** 2003). Deformation surpassing the yield point could disrupt top layers of biofilm, or its EPS matrix (cohesive failure), or could completely remove the biofilm (adhesive failure). If deformation is still in the plastic range but below the yield point, biofilm is expanded but not removed (Busscher *et al*.** 2003). Disruption of the top layers or EPS matrix or expansion of the biofilm eases irrigant penetration in the biofilm and could therefore enhance the chemical effect of irrigants (He *et al*.** 2013). Moreover, a disruption of the biofilm matrix could leave ‘footprints’ in the post‐treatment remaining biofilm, which may facilitate or impede adhesion of microorganisms, thereby influencing reorganization of the biofilm (Busscher *et al*.** 2003).

Microorganisms in the root canal live in a biofilm state and are consequently protected by a matrix structure (EPS). Antimicrobials need to diffuse into the EPS matrix to enhance an effect. Therefore, the potential of EPS penetration, disruption and killing of the microorganisms is all inherently related. Furthermore, antimicrobials can alter the mechanical properties of EPS, which may be explained by an effect on the EPS network formation (Korstgens *et al*.** 2001). This alteration can directly influence the removal of the biofilm (Brindle *et al*.** 2011) and influence the data.

NaOCl removed significantly more biofilm from the lateral canal‐ and isthmus‐like structure than the inert active control. This is in contrast to an earlier study using the same model (Pereira et al. 2020b). However, in that study, irrigant exposure time was 30 s and the flow was constant. In the present study, the irrigants were applied during 30 or 15 s (flow rate 0.05 or 0.1 mL s^−1^) after which the irrigants were left in the root canal for 5–15 min without irrigant flow depending on the refreshment schedule. This indicates that longer than 30 s was needed for a significant biofilm removal to occur through chemical‐driven diffusion when the biofilm is dense and the contact surface small. However, no difference in biofilm removal was seen between 5, 7.5 and 15 min, indicating that after 5 min no significant removal occurred.

Refreshment of irrigant had no influence on biofilm removal from both the isthmus and the lateral canal‐like structures. Apparently, application of fresh NaOCl close to the biofilm did not improve biofilm removal. Possibly because the refreshment was performed after 5 min and the results reveal that 5 min were enough for diffusion. This is in line with the conclusion of Macedo *et al*.* *(2010, 2014b) that refreshment will not compensate for the use of a lower concentration of NaOCl. Possibly in the present model, the amount of free chlorine present in the root canal was enough to sustain diffusion. Furthermore, without refreshment approximately 60% of the biofilm had been removed. Most likely the removal of the last part of the biofilm is difficult and requires other mechanisms. De Beer *et al*. (1994) studied the penetration of chlorine through a *Pseudomonas aeruginosa/Klesiella pneumoniae* biofilm and concluded that diffusion into the biofilm is a slow process, chlorine is reduced in the matrix, diffusion rate depends on the concentration, there is a diffusion‐reaction mechanism and large variation due to local differences (highly resistant spots). These highly resistant spots are associated with a higher cell density with subpopulations with a higher reducing capacity per cell. Furthermore, these spots have a higher density of EPS with a greater reducing potential. Combined with a fast regrowth after an antimicrobial treatment, these highly resistant spots are serious threats. In the biofilm used in this actual root canal model, these highly resistant spots have been identified and impede removal (Busanello *et al*.** 2019).

Mass transfer inside a biofilm occurs by convection and (mainly) diffusion (De Beer *et al*.** 1994). The surface‐averaged relative effective diffusion coefficient (*D*_rs_) decreases from the top of the biofilm towards the bottom. The *D*_rs_ profiles differ for biofilms of different ages and generally decrease over time. In addition, different biofilms showed similar *D*_rs_ profiles near the top of the biofilm but different *D*_rs_ profiles near the bottom of the biofilm (Renslow *et al*.** 2010). This bottom layer also determines the attachment to the surface, in the present case PDMS, and is normally the most difficult to remove (Derlon *et al*.** 2008).

Higher flow rate removed significantly more biofilm only from the isthmus‐like structure. Earlier studies using the same root canal model also demonstrated that flow rate had a higher impact on biofilm removal from an isthmus‐like structure than a lateral canal‐like structure ((Pereira et al. 2020a,b) Macedo *et al*. 2014b). The distribution of irrigant will depend on the anatomy of the root canal (Gulabivala *et al*.** 2005). Because the opening of the isthmus is larger than the lateral canal, the former allows a steady stream of irrigant, favouring a slow and steady viscous biofilm removal after an initial rapid and unsteady removal (Jiang *et al*.** 2010, Verhaagen *et al*.** 2014) whereas the latter favours removal by pieces (Macedo *et al*.** 2014b).

In the group with 2 refreshments, biofilm removal was analysed after each 5‐min time interval. After every step, significantly more biofilm was removed. Since no significant difference was seen between exposure time of 5 and 15 min, this difference has to be caused by the flow rate of the irrigant. However, the total amount of biofilm removed after 15 min was not significantly more than the group without irrigant refreshment.

Biofilm research has its difficulties, and every methodology has its own drawbacks (Swimberghe *et al*.** 2019). Notwithstanding the fact that the biofilms were grown under standardized conditions all isthmus and lateral canal‐like structures contained different volumes of biofilm. OCT enables a longitudinal evaluation of biofilm allowing measurements at different time points using the biofilm as its own control. Nonetheless, this variation in volume can cause a relatively large standard deviation.

Only a few studies have used a cell‐rich biofilm with viscoelastic properties of an oral biofilm and a more realistic surface contact irrigant‐biofilm. It seems that biofilm removal from the last part of the biofilm is more difficult than expected from earlier work. This could be related to the resistant spots or the specifics location and accessibility. However, it correlates better with clinical outcome of root canal treatment where it seems to be impossible to remove biofilm completely using NaOCl as an irrigant. The recalcitrance of the biofilm and the complex root canal anatomy seems to be crucial. Furthermore, in the clinical situation, lateral morphological features in the root canal are probably covered with dentine debris produced during instrumentation of the root canal (Paqué *et al*.** 2012) further hampering biofilm removal (Arias‐Moliz *et al*.** 2016).

## Conclusions

In this typical root canal model with lateral morphological features, refreshment of irrigant did not improve biofilm removal suggesting the root canal itself contained a sufficient amount of reactive molecules for biofilm removal. NaOCl removed more biofilm from the lateral canal and isthmus structure and a higher flow rate removed more biofilm from the isthmus‐like structure. There was always remaining biofilm left after the irrigation procedures.

## Conflict of Interest

Dr. Thais Pereira reports grants from Coordenação de Aperfeiçoamento de Pessoal de Nível Superior – CAPES and Abel Tasman Talent Program. The other authors have stated explicitly that there are no conflicts of interest in connection with this article.
